# Optimized river diversion scenarios promote sustainability of urbanized deltas

**DOI:** 10.1073/pnas.2101649118

**Published:** 2021-06-28

**Authors:** Andrew J. Moodie, Jeffrey A. Nittrouer

**Affiliations:** ^a^Department of Earth, Environmental and Planetary Sciences, Rice University, Houston, TX 77005;; ^b^Department of Civil, Architectural and Environmental Engineering, University of Texas at Austin, Austin, TX 78712

**Keywords:** delta sustainability, river deltas, avulsion, river diversion

## Abstract

Socioeconomic viability of fluvial-deltaic systems is limited by natural processes of these dynamic landforms. An especially impactful occurrence is avulsion, whereby channels unpredictably shift course. We construct a numerical model to simulate artificial diversions, which are engineered to prevent channel avulsion, and direct sediment-laden water to the coastline, thus mitigating land loss. We provide a framework that identifies the optimal balance between river diversion cost and civil disruption by flooding. Diversions near the river outlet are not sustainable, because they neither reduce avulsion frequency nor effectively deliver sediment to the coast; alternatively, diversions located halfway to the delta apex maximize landscape stability while minimizing costs. We determine that delta urbanization generates a positive feedback: infrastructure development justifies sustainability and enhanced landform preservation vis-à-vis diversions.

## Significance

Urbanization is widely considered to limit river-delta sustainability by inhibiting natural land-building processes that are necessary to mitigate the detrimental effects of sea-level rise and coastal storm surge. In response, billions of dollars have been spent globally to engineer river diversions that distribute water and sediment to coastlines; however, the broader cost and benefits of locating such infrastructure have not been evaluated. Here, we show that the optimal location for diversions depends on coevolution of natural river processes and tradeoffs between societal benefit and diversion cost. Our findings provide a quantitative description of nature-based solutions and will inform decision-making frameworks that seek sustainable and equitable delta and coastline landscape management strategies.

Deltaic environments are critical for societal wellbeing because these landscapes provide an abundance of natural resources that promote human welfare (1, 2). However, the sustainability of deltas is uncertain due to sea-level rise (3, 4), sediment supply reduction (4–6), and land subsidence (7, 8). Additionally, river avulsion, the process of sudden channel relocation (9, 10), presents a dichotomy to delta sustainability: the unanticipated civil disruption associated with flooding brought by channel displacement is at odds with society’s desire for landscape stability, yet channel relocation is needed to deliver nutrients and sediment to various locations along the deltaic coastline (11, 12). Indeed, for many of the world’s megadeltas, channel engineering practices have sought to restrict channel mobility and limit floodplain connectivity (13, 14), which in turn prevents sediment dispersal that is necessary to sustain deltas; as a consequence, land loss has ensued (15). Despite providing near-term stability (13–15), engineering of deltaic channels is a long-term detrimental practice (11, 15–17).

**Fig. 1.**
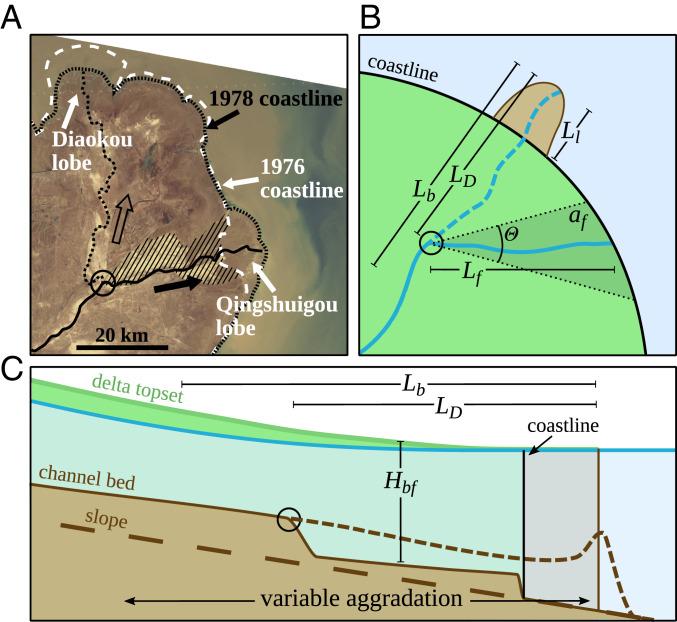
(*A*) Satellite image of Yellow River delta (Landsat, 1978) showing coastline response to a diversion in 1976 at the open circle, which changed the channel course from the north (Diaokou lobe) to the east (Qingshuigou lobe) and produced flooding over the stripe-hatched area (30). (*B* and *C*) Planform view (*B*) and along-channel cross-section view (*C*) of conceptual model for numerical simulations and societal benefit formulation. In the diagrams, a diversion at $L_{D}\approx 0.8L_{b}$ floods an area ($a_{f}$) defined by $L_{f}$ and θ, diverting sediment away from the deltaic lobe (with length $L_{l}$). Aggradation of the former channel bed (dashed line) is variable; hence, diversion length influences the propensity for subsequent avulsion setup.

To maximize societal benefit, measures that promote delta sustainability must balance engineering infrastructure cost and impact on delta morphology with benefits afforded by maintaining and developing deltaic landscapes (1, 2, 11, 12, 16–19). For example, channel diversions, costing millions to billions of dollars (20–22), are now planned worldwide to both prevent unintended avulsions and ensure coastal sustainability through enhanced sediment delivery (e.g., Fig. 1*A*) (20, 21, 23–26).

In this article, we consider the benefits and costs of such engineered river diversions and determine how these practices most effectively sustain deltaic landscapes, by assessing optimal placement and timing for river diversions. Addressing these points requires combining two modeling frameworks: a morphodynamic approach—evolving the landscape over time and space by evaluating the interactions of river fluid flow and sediment transport—and a decision-making framework (21, 22, 27, 28). The former simulates deltaic channel diversions by assessing the nonlinear relationships between channel diversion length ($L_{D}$) and the frequency (timing) of avulsions ($T_{A}$), while the latter incorporates a societal benefit model that approximates urbanization by considering the cost of flooding a landscape that would otherwise generate revenue. The aim is to optimize timing and placement of channel diversions, by giving consideration to morphodynamic operations and societal wellbeing. Interestingly, optimal societal benefit indicates that urbanization justifies enhanced sustainability measures, which contradicts existing paradigms that label development and sustainability mutually exclusive (3, 7, 12). Ultimately, the societal benefit model should be an integrated component in decision-making frameworks. This will help locate diversions and promote sustainable and equitable decisions considering historical, ethical, and environmental contexts for river management decisions (29).

## Diversions on River Deltas

Channel avulsions occur naturally as a consequence of sediment aggradation on the river bed (i.e., avulsion setup) (9, 31), in conjunction with a flood event that generates a levee-breaching flow (i.e., avulsion trigger) (10). As a result, water leaves a constricted channel and establishes an alternative route over the floodplain. In delta settings, hydrodynamic conditions arise (i.e., slowing flow velocity) that lead to variable along-channel sediment aggradation (Fig. 1*C*) (32). An abundance of research has demonstrated that the location where sedimentation is maximized is scalable across deltas of all size; defined as the backwater length ($L_{b}$), the upstream extent of nonuniform flow in the delta is the most important morphodynamic parameter of deltaic systems (33). Thus, backwater sedimentation locates avulsions (i.e., avulsion length $L_{A}\approx L_{b}$) and the long-term (average) avulsion location ($L_{A,0}$) (30, 34). Furthermore, by evaluating sediment aggradation rates, it is possible to assess a long-term (average) avulsion timescale ($T_{A,0}$). As a result of an avulsion, a new deltaic lobe builds.

The classic case study of a natural system that has been used to bolster the above-referenced studies and spatiotemporal relationships is the Yellow River delta (China) (30), where avulsions occur naturally every decade (23), making this system one of the most active megadeltas worldwide. Nowadays, avulsions for this system are prevented by superseding channel diversions, usually implemented around the average avulsion timescale ($T_{A,0}$, ∼10 y) (35, 36). However, these diversions are not designated spatially with respect to the avulsion length scale ($L_{A}$, ∼60 km) (35, 37).

Engineered diversions create a new channel on the delta floodplain from the diversion location to the coast that establishes a depth and width in equilibrium with upstream sediment transport and water flow conditions; as a result, diversion channel volume scales primarily with diversion length. The new channel is prone to variable sedimentation due to backwater hydrodynamics (Fig. 1*C*), which, coupled with channel volume, introduces a nonlinear influence on sedimentation and the propensity for avulsion setup.

To model this, we start with the null hypothesis that spatially uniform deposition scales the time to subsequent avulsion linearly with diversion length: ${T_{A}}^{*}\approx {L_{D}}^{*}$, where the relation is cast in dimensionless time (${T_{A}}^{*}=T_{A}/T_{A,0}$) and length (${L_{D}}^{*}=L_{D}/L_{b}$). Alternatively, a diversion upstream of the backwater segment could nonlinearly increase time to the next avulsion. We let ${T_{A}}^{*}={\left({L_{D}}^{*}\right)}^{n}$, where 0<n<1, choosing n=1/2 (i.e., ${T_{A}}^{*}=\sqrt{{L_{D}}^{*}}$). This simple form is bolstered by delta channel morphodynamics that establish the likely avulsion location (30, 32–34): the function intersects the origin (${T_{A}}^{*}=0$ when ${L_{D}}^{*}=0$) and sets the dimensionless time to subsequent avulsion equal to the long-term avulsion timescale for a diversion at the natural avulsion location (${T_{A}}^{*}=1$ when ${L_{D}}^{*}=1$).

### Modeling Diversions.

We used a numerical delta model (30) to simulate along-channel flow and sediment transport (*Materials and Methods* and *SI Appendix*, section A). One-dimensional flow is joined with a two-dimensional, radially symmetric delta-building model that builds a lobe (Fig. 1*B*). An avulsion occurs in the model when the channel bed aggrades to a defined proportion of the bankfull depth (i.e., a threshold superelevation coefficient, β) (31). The model was run for several natural avulsion cycles and halted just before another avulsion (i.e., at β), establishing the initial conditions for a diversion model that is intended to approximate the onset of delta lobe building after an avulsion. We varied β between 0.4 and 0.5, which scales avulsions $L_{A,0}\approx L_{b}$ and the characteristic lobe length $L_{L}\approx 0.5L_{b}$ (30).

**Fig. 2.**
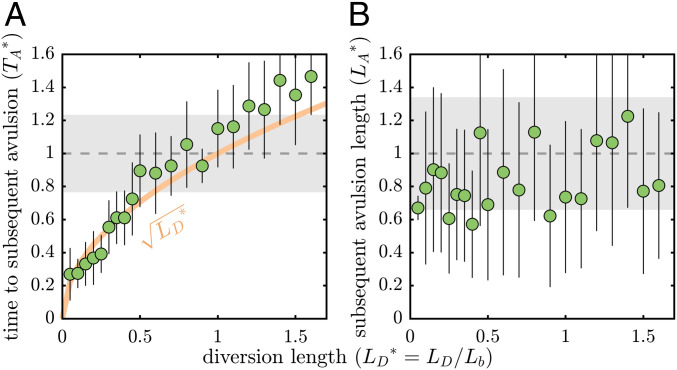
(*A* and *B*) Dimensionless time to (*A*) and length of (*B*) a subsequent avulsion following artificial diversions. Points are Yellow River delta simulation mean and 1σ. Gray shading shows long-term average avulsion time- and length-scale 1σ interval. Time to subsequent avulsion (${T_{A}}^{*}$) increases nonlinearly with artificial diversion length, yet superelevation is reached at approximately ${L_{A}}^{*}=1$ in all simulations.

Diversions were simulated from ${L_{D}}^{*}=0.05$ to 1.6, whereby a channel pathway is established as one bankfull flow depth below the delta topset elevation (31) from the diversion site to the coastline ($L_{f}$; Fig. 1*B* and *SI Appendix*, section A). A diversion was simulated with a constant slope, by connecting the upstream channel and the new channel pathway (30). Simulations were run until channel superelevation was reached, just before a natural avulsion. Ten simulations were conducted at each diversion length ($L_{D}$; Fig. 2).

Avulsions occurred at approximately the long-term average avulsion timescale ${T_{A}}^{*}\approx 1$ for ${L_{D}}^{*}=1.0$ (Fig. 2*A*). Interestingly, the time to a subsequent avulsion increased nonlinearly with diversion length (Fig. 2*A*). For example, diversions set at ${L_{D}}^{*}=0.1$ were followed by superelevation conditions after a disproportionately long time (${T_{A}}^{*}\approx 0.3$). The location where superelevation conditions were reached (${L_{A}}^{*}$, subsequent avulsion location without intervention) was approximately constant (${L_{A}}^{*}\approx 1$) and within 1σ of the long-term average avulsion timescale for all diversion lengths (Fig. 2*B*). An important outcome is that the simulations are similar to ${T_{A}}^{*}=\sqrt{{L_{D}}^{*}}$ (Fig. 2*A*).

### Evolution of Channel Bed Elevation Following Diversions.

**Fig. 3.**
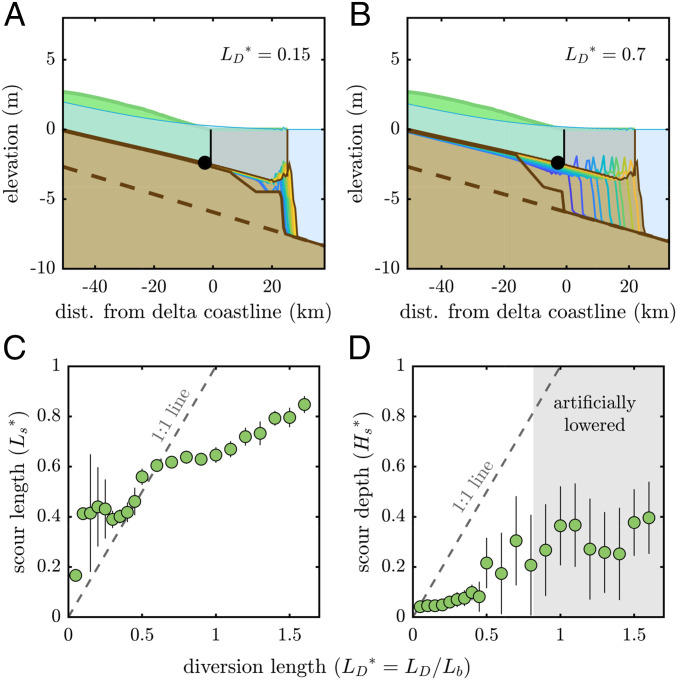
Bed erosion measured in diversion simulations. (*A* and *B*) Representative model simulation results depicting topographic change following diversions at ${L_{D}}^{*}=0.15$ and ${L_{D}}^{*}=0.7$. Blue to yellow lines denote time progression, black circle is location where superelevation is achieved, and additional styling is consistent with Fig. 1*C*. (*C* and *D*) The erosional scour length (*C*) and depth (*D*) both increased with diversion length. Scour length was demarcated by the *e*-folding depth upstream from the location of maximum erosion depth (38) and increased sharply for diversions longer than ${L_{D}}^{*}=0.5$. Scour depth was measured at the location of the subsequent avulsion (${L_{A}}^{*}$) and increased significantly for diversions longer than ${L_{D}}^{*}=0.5$.

Following diversions, the channel bed near the diversion site is eroded (Fig. 3 *A* and *B* and *SI Appendix*, section A). This generates accommodation space for sediment and prolongs the time until a subsequent avulsion. The channel bed and delta lobe long profiles evolve to a consistent configuration when superelevation is achieved, irrespective of the diversion location (Fig. 3 *A* and *B*).

The along-channel scour length ($L_{s}$; ${L_{s}}^{*}=L_{s}/L_{b}$) was identified by determining the erosion depth *e*-folding distance upstream from the location of maximum erosion (38). The scour depth ($H_{s}$; ${H_{s}}^{*}=H_{s}/H_{bf}$, where $H_{bf}$ is bankfull flow depth) was determined at the subsequent avulsion location. Channel bed scour length and depth increased unsteadily with diversion length (Fig. 3 *C* and *D*). Second-order scour events were noted, arising due to hydrodynamic drawdown (30, 33, 38).

A newly diverted channel path is steeper than the channel path upstream of the diversion, leading to enhanced sediment transport capacity (30, 34, 39) and generating an upstream migrating degradational wave (38). The scour length is limited by the duration of erosion, which is dependent on diversion length (${L_{D}}^{*}$). This finding is unique, as other models do not show a dependence on channel length (38). The time required to reach superelevation is not constant for all diversion lengths, indicating that morphodynamic operations inform the value of diversions from a per-unit-cost basis.

## Delta Management and Societal Benefit

For delta management strategies to be effective, financial costs and societal benefits must be optimized. Such a framework requires combining geomorphic considerations with representations of societal wellbeing (18). Herein, we focus on the subaerial landscape of the delta topset to formulate an expression of societal wellbeing in terms of productivity and liabilities, integrated over the delta area and over one avulsion cycle (i.e., from one avulsion or diversion to the next).

First, to understand the benefit derived from deltaic land use, we refer to an axisymmetric delta topset with an area $a_{d}=\left(\pi /4\right){L_{d}}^{2}$ (Fig. 1*B*) (30), where $L_{d}$ is the distance from the delta apex to the radially averaged shoreline, and π≈3.1415. The area flooded by an avulsion event is $a_{f}\approx \tan\left(\theta /2\right){L_{f}}^{2}$, where θ is an opening angle from the levee breach, and $L_{f}$ is the distance from levee breach to shoreline (i.e., the flooding length). The cost of flooding per-unit time is determined as $a_{f}C_{f}$, where $C_{f}$ is the flooding damage rate per-unit area, per-unit time ($/$L^{2}\cdot$T). The land area of a delta that remains unflooded over the avulsion cycle duration produces revenue per-unit area, per-unit time ($C_{p}$).

Additionally, delta lobe progradation rate is $r_{l}$, such that lobe area over time is $a_{l}=L_{l}B_{l}=r_{l}tB_{l}$, where $L_{l}$ is lobe length, $B_{l}$ is lobe width, and t is time. Geometric scaling, physical experiments, and numerical modeling suggest that lobe length at the time of avulsion is proportional to channel bed superelevation and the backwater length, such that $L_{l}\approx r_{l}T_{A}\approx \beta L_{b}$ (30, 39, 40). Thus, lobe revenue per-unit time is given by $a_{l}C_{l}$, where $C_{l}$ is the lobe-land revenue per-unit area, per-unit time.

Channel relocation may be natural (avulsion) or artificial (engineered diversion). Either circumstance incurs indirect costs associated with land flooding. Avulsions have no direct cost, whereas diversions are composed of a fixed cost ($C_{D,x}$) and a variable cost that depends on diversion length ($C_{D,v}$):

$$C_{D}=C_{D,x}+L_{f}C_{D,v}.$$ [1]

We assume that $C_{D,v}$ incorporates costs associated with acquiring land and trenching a new channel and that $C_{D,x}$ comprises costs associated with construction of the diversion and relocating industry. Annual operation and maintenance costs are assumed to be negligibly small compared to the expense of construction.

We assume a single levee-breaching flood per avulsion cycle and define an indicator function (I(t)) that equals 1 in the flood event time interval and 0 otherwise. Then, our formulation for societal benefit (Π) over the duration of the avulsion cycle is given as

$$\begin{array}{cc} \Pi =C_{p} & \left(a_{d}-a_{f}\right)T_{A}-C_{D}+\\ & \int^{T_{A}}r_{l}tB_{l}C_{l}+C_{p}a_{f}\left(1-I\left(t\right)\right)-C_{f}a_{f}I\left(t\right)\;dt. \end{array}$$ [2]

Terms on the right side of Eq. **2** describe (in order)

• benefit from use of unflooded delta topset area,
• cost of diversion construction,
• benefit from use of deltaic lobe land,
• benefit from use of flooded delta topset area, and
• cost of flooding over flooded delta topset area.

Eq. **2** is nondimensionalized (*SI Appendix*, section B) to give the dimensionless societal benefit per unit time of a managed delta:

$$\begin{array}{cc} \lambda_{\Pi ,diversion}=1+\frac{2\beta }{\pi } & R_{l}\lambda_{l}-\frac{\lambda_{D}}{{T_{A}}^{*}}\left[1+\alpha {L_{f}}^{*}\right]-\\ & \frac{\left[1+\lambda_{f}\right]}{{T_{A}}^{*}}\tan\left(\theta /2\right){{L_{f}}^{*}}^{2}, \end{array}$$ [3]

where $\lambda_{D}$, $\lambda_{f}$, and $\lambda_{l}$ are the ratio of diversion cost, flooding cost, and lobe land-use benefit to delta land-use benefit; α is the ratio of variable diversion cost per-unit length to fixed diversion cost; $R_{l}=B_{l}/L_{b}$ is dimensionless lobe width; and ${L_{f}}^{*}$ is the length of flooded land area. Terms on the right side of Eq. **3**, following the constant, represent societal benefit from lobe land, diversion cost, and flooding cost, respectively; additional terms and parameters in Eq. **2** are eliminated by integration or nondimensionalization (*SI Appendix*, section B).

Our societal benefit formulation assumes 1) only one flooding event per avulsion cycle $T_{A}$, 2) flooded area length (${L_{f}}^{*}$) is fixed for diversions on the delta lobe, and 3) flooding damage unit cost is equal on the lobe and delta (i.e., $\lambda_{f}$ is fixed regardless of ${L_{D}}^{*}$). These assumptions, and linear transformations of parameters (*SI Appendix*, section B), nondimensionalize the framework and render our formulation universal and therefore applicable to delta systems globally. Notably, the ${L_{f}}^{*}$ formulation is an important control on the behavior of Eq. **3**, because it prevents exponential increase in flooding area with increasing ${L_{D}}^{*}$ until ${L_{D}}^{*}>\beta$. Importantly, with a suitable relationship predicting ${T_{A}}^{*}$ given ${L_{D}}^{*}$, Eq. **3** depends only on ${L_{D}}^{*}$ and seven dimensionless parameters $\lambda_{f}$, $\lambda_{l}$, $\lambda_{D}$, α, β, tan(θ/2), and $R_{l}$.

The dimensionless economic parameters ($\lambda_{f}$, $\lambda_{l}$, and $\lambda_{D}$) have practical meaning. The parameter $\lambda_{f}$ measures the ratio of asset value lost to flooding to yearly revenue and is expected to be of order $10^{0}$ and ≥1. $\lambda_{f}$ increases beyond unity with increasing urbanization of a delta; flooding farmland destroys one unit-time crop ($\lambda_{f}=1$), whereas flooding an urbanized delta destroys infrastructure expected to yield revenue over multiple unit times ($\lambda_{f}\gg 1$). The value for $\lambda_{l}$ captures the value of coastal land relative to delta land and is expected to be of order unity. For example, $\lambda_{l}$ may exceed unity for the Mississippi River delta, where hurricane impacts and storm damage costs are common and therefore lower the time-integrated land-use revenue ($C_{p}$) relative to lobe revenue ($C_{l}$) (1, 27). $\lambda_{D}$ ratios the artificial diversion cost to the land-use revenue and incorporates ecosystem services value (41), such as unique ecological habitats. The value of $\lambda_{D}$ is conceptually related to cost of capital, because each relates investment cost to future cash flow (42), and so $\lambda_{D}$ is expected to scale with societal development (43) and is inversely related to delta size (via $L_{b}$). $\lambda_{D}$ is difficult to constrain, but is expected to be of order $10^{-4}$ to $10^{-1}$ (*SI Appendix*, section C).

In the following sections, we examine how ${L_{D}}^{*}$ affects societal benefit for various relationships between ${T_{A}}^{*}$ and ${L_{D}}^{*}$. We additionally explore how cost parameters ($\lambda_{f}$, $\lambda_{l}$, $\lambda_{D}$) affect the optimal location for artificial diversions. We assessed uncertainty in the societal benefit formulation with Monte Carlo sampling from probability distributions for the cost coefficient (α), dimensionless parameters reflecting physical attributes of the delta system (β, θ, and $R_{l}$), and cost parameters not systematically varied. We defined α∼U(0.01,0.05), β∼U(0.4,0.6), θ∼Γ(2,6), $R_{l}∼N\left(0.22,0.08\right)$, $\lambda_{f}∼N\left(2,0.1\right)$, $\lambda_{l}∼N\left(1,0.1\right)$, $\lambda_{D}∼N\left(0.01,0.03\right)$ (*SI Appendix*, section C); normal distributions are truncated in [0,∞).

### Utility of Various Delta Management Strategies.

**Fig. 4.**
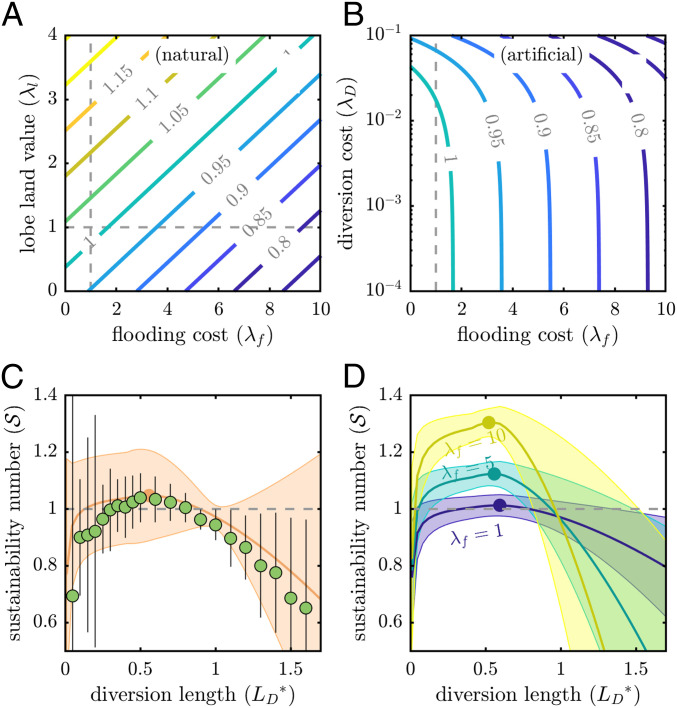
(*A* and *B*) Societal benefit derived from natural delta evolution (*A*) (Eq. **4**) and from artificial diversions (*B*) (Eq. **3**) at $L_{D}*=1$ for parameter range of flooding damage ($\lambda_{f}$) and lobe value ($\lambda_{l}$) or diversion cost ($\lambda_{D}$). (*C*) Sustainability number (Eq. **5**) evaluated for function $T_{A}*=\sqrt{L_{D}*}$ and morphodynamic simulations for Yellow River delta system, with shaded region and error bars representing 95% confidence interval from Monte Carlo parameter sampling. (*D*) Sustainability number for function $T_{A}*=\sqrt{L_{D}*}$, evaluated for systematic change in flooding cost relative to land-use revenue ($\lambda_{f}$), showing that the sustainability increases for more urban deltas.

Natural avulsions have no direct cost, so we set $\lambda_{D}=0$. We expect that for avulsions $L_{D}=L_{A}=L_{b}$ (i.e., ${L_{D}}^{*}={L_{A}}^{*}=1$) (34), and ${T_{A}}^{*}=1$. This reduces Eq. **3** to

$$\lambda_{\Pi ,natural}=1+\frac{2\beta }{\pi }R_{l}\lambda_{l}-\tan\left(\theta /2\right){\left(1-\beta \right)}^{2}\left[1+\lambda_{f}\right],$$ [4]

which is a function of only dimensionless parameters and is linear in $\lambda_{f}$, $\lambda_{l}$, and $R_{l}$. We evaluated the natural delta evolution societal benefit (i.e., Eq. **4**) for a range of flooding costs and lobe values, while fixing other parameter distributions to their expected value (Fig. 4*A*). The model behavior is intuitive insofar that societal benefit decreases as flooding costs increase and that benefit increases when lobe land area has a larger value.

Similarly, we examined the change in societal benefit as a function of artificial diversion costs and flooding costs (i.e., Eq. **3** and Fig. 4*B*). This space is linear in the parameters we systematically varied ($\lambda_{f}$ and $\lambda_{D}$) and demonstrates that raising artificial diversion costs lowers dimensionless benefit to society. However, the diversion cost impacts the societal benefit minimally when $\lambda_{D}<10^{-2}$, implying that for larger deltas, where $\lambda_{D}\to 0$, the precise value of diversion cost is largely irrelevant.

Natural delta evolution provides an important baseline for assessing sustainability, whereby engineering strategies with societal costs exceeding benefits are economically unsustainable. We thus define the sustainability number S,

$$S=\frac{\lambda_{\Pi ,diversion}}{\lambda_{\Pi ,natural}},$$ [5]

evaluated for a given set of dimensionless parameters and variables. A value greater than unity indicates a delta management strategy that provides a net benefit to society.

We evaluated the sustainability number (S, Eq. **5**) for artificial diversions from ${L_{D}}^{*}=0.05$ to 1.6, using the function ${T_{A}}^{*}=\sqrt{{L_{D}}^{*}}$ and delta morphodynamic simulation results; uncertainty was quantified with Monte Carlo sampling. In all evaluations, S is maximized for diversions at ${L_{D}}^{*}=0.5$ to 0.7 (Fig. 4*C*). Furthermore, the optimum persists despite changes in all dimensionless parameters and is independent of the relationship between ${T_{A}}^{*}$ and ${L_{D}}^{*}$ (*SI Appendix*, section B); although the curves shift vertically with changing cost parameters, and the optimum ${L_{D}}^{*}$ shifts laterally with change in β (*SI Appendix*, section B).

**Fig. 5.**
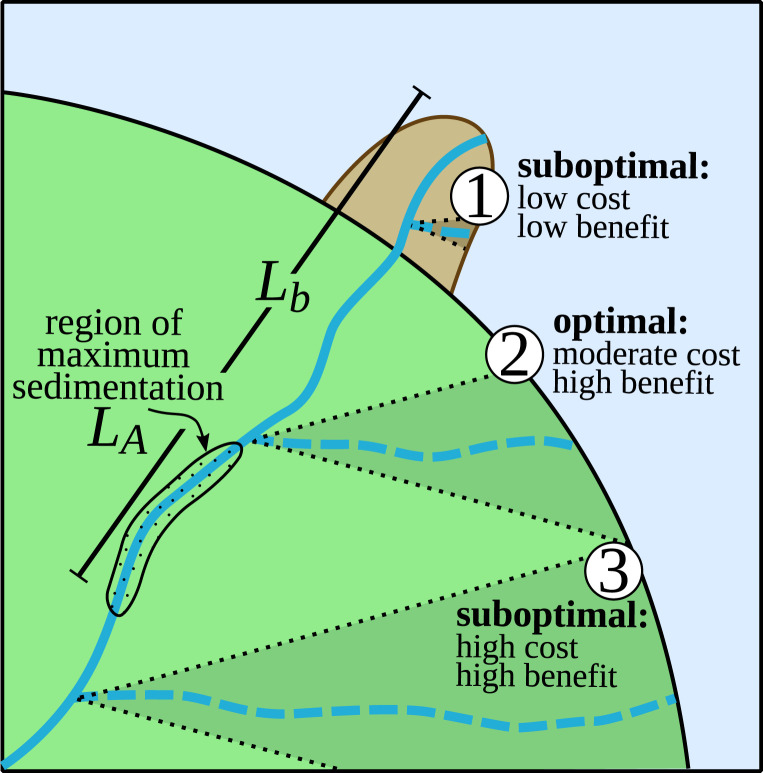
Conceptual summary depicting three potential diversion scenarios. The natural avulsion location ($L_{A}$) is determined by the region of maximum sedimentation within the delta, near the upstream extent of the backwater length ($L_{b}$). Diversion 1 cost is low, but does not lower the channel bed at the natural avulsion location or increase the time until a natural avulsion, and so it provides very little societal benefit. In contrast, diversion 2 has a moderate cost, but creates an upstream-migrating scour wave that lowers the channel bed in the region of maximum sedimentation, significantly increasing the time to a natural avulsion, and so provides high societal benefit. Diversion 3 bypasses the region of maximum sedimentation entirely, and so it provides high societal benefit, but diversion costs are too high to be justifiable.

The optimum S arises due to the tradeoff between increasing flood and diversion costs and the increasing time over which both costs are amortized (i.e., ${T_{A}}^{*}$ in Eq. **3**) (Fig. 5). Diversions near the river outlet incur relatively high costs that are amortized over only ${T_{A}}^{*}\ll 1$, thus lowering the sustainability number (i.e., S≪1). Diversion cost increases $\propto {{L_{D}}^{*}}^{1}$, but flooding cost increases $\propto {{L_{D}}^{*}}^{2}$ (Eq. **3**), so while the increase in ${T_{A}}^{*}$ outpaces diversion cost increase (i.e., lowering the amortized diversion cost →0), the flooding cost increase outpaces the ${T_{A}}^{*}$ increase, leading to ever-higher flooding costs with increasing ${L_{D}}^{*}$. The flooding area (${a_{f}}^{*}$) formulation prevents flooding cost increase until ${L_{D}}^{*}>\beta$, so the optimum location of diversions arises for ${L_{D}}^{*}=0.5$ to 0.7. To summarize, the societal benefit decreases for longer artificial diversions (${L_{D}}^{*}≳1$), because flooding cost becomes large per-unit increase in time (Fig. 5); this is consistent with the economic concept of diminishing returns.

## Artificial Diversions Are Sustainable

Artificial diversions enhance delta sustainability. While conventional studies assert that urbanization limits the feasibility of long-term sustainability of river deltas (7, 12), our assessment of the societal benefit provided by artificial diversions indicates that river-delta urbanization increases the sustainability number. Importantly, this is a positive feedback, whereby increased urbanization makes artificial diversions more attractive despite higher costs. This implication is best realized in situations where retreat from a delta is not practical (e.g., The Netherlands) or would destroy centuries worth of cultural history (e.g., Louisiana). A positive urbanization feedback means that these societies do not need to retreat from deltas.

### Urbanization and Sustainability: A Positive Feedback.

$\lambda_{f}$ is a proxy for delta urbanization, because more urban deltas have capital investment and infrastructure that provide revenue over time, rather than in a single unit time (i.e., $\lambda_{f}>1$). We examined the change in sustainability number (S) for a systematic increase in flooding costs ($\lambda_{f}$; Fig. 4*D*). The gradient of sustainability number change with ${L_{D}}^{*}$ is amplified for larger $\lambda_{f}$, and the optimal S increases with increasing $\lambda_{f}$ (Fig. 4*D*).

Optimal S increases with $\lambda_{f}$ because the societal benefit obtained from natural delta evolution decreases faster than for artificial diversions. Societal benefits from natural and artificial delta management both decrease with larger $\lambda_{f}$, and benefit from diversions at ${L_{D}}^{*}=1$ is always less than the natural benefit, because of diversion construction costs (*SI Appendix*, section B). However, artificial diversions ${L_{D}}^{*}⪅0.7$ reduce flooding area with respect to natural delta evolution and increase the time to a subsequent avulsion beyond a factor of 1:1 (i.e., nonlinearly; Fig. 4*D*). As a result, the sustainability number is amplified as flooding costs increase. The values of α, β, θ, $\lambda_{l}$, $\lambda_{D}$, or $R_{l}$ do not impact the net increase in sustainability number with urbanization (*SI Appendix*, section B).

Urbanization creates a positive feedback on sustainability because it enables increasingly expensive projects that seek to sustain the delta landscape and further benefit the system and society. This is in contrast to a significant body of research that has painted urbanization as a limit to delta sustainability (3, 7, 12, 19), which often limits sustainability to preserving pristine landscapes and ecosystems unadulterated by human intervention. We challenge this convention, insofar that it places unrealistic constraints on delta management strategies. Instead, our work highlights the importance of taking a holistic view to delta management, whereby a strategy exists that maximizes societal benefit under realistic socioeconomic constraints. Our societal benefit formulation demonstrates that there is a positive feedback that argues in favor of continued use and development of deltas, because engineering works, specifically via artificial diversions that maintain a topset footprint, are economically viable and foster the sustainability of delicate deltaic coastal systems.

### Natural Processes Influence Optimal Decision-Making.

The variable magnitude of erosion immediately following a diversion demonstrates the importance of a comprehensive morphodynamic assessment (Fig. 5). In the hypothetical case where time to avulsion (${T_{A}}^{*}$) is equal for all diversion lengths (${L_{D}}^{*}$), the diversion length that maximizes societal benefit is that which minimizes the direct cost of the diversion (i.e., ${L_{D}}^{*}\to 0$). However, ${T_{A}}^{*}$ scales with ${L_{D}}^{*}$, because an artificial diversion near the river outlet (${L_{D}}^{*}⪅0.5$) does not induce significant scour and lower the channel bed at the natural avulsion location (${L_{A}}^{*}=1$; Fig. 3*D*). In contrast, diversions farther upstream lower the channel bed at ${L_{A}}^{*}$, so while shorter diversions are attractive from a cost perspective, the delta system morphodynamic behavior does not conform. As a result, multiple short diversions are needed to prevent an avulsion for the duration ${T_{A}}^{*}=1$, resulting in higher overall cost than a single diversion farther upstream (Fig. 4*C*). Importantly, a decision-making framework that ignores geomorphic constraints (i.e., scaling between ${T_{A}}^{*}∼{L_{D}}^{*}$) could select a suboptimal diversion location (Fig. 5).

### Delta Management Decision-Making.

Our findings emphasize the importance of a long-term perspective when evaluating delta management strategies, in particular when considering sites for artificial diversions (18). The unsustainable nature of diversions near the river outlet provides policy makers with guidance to evaluate larger-scale diversions.

The practical meaning of dimensionless cost parameters elucidates how optimal decisions change in response to evolving societal values. For example, asset investment leads to increased revenue generated from land use (i.e., increasing $\lambda_{f}$), which alters incentives for the society to actively manage a deltaic system. Freshwater and nutrient supply via channel diversion can bolster ecosystem health (44); ecosystem service value is correlated with societal development and is reflected in our formulation by increasing land-creation value ($\lambda_{l}$) or the diversion cost ($\lambda_{D}$).

There are several assumptions in our model that may limit direct application to any specific delta. We neglect space-filling constraints of deltaic lobe building (30, 45). For example, diversion outlets should be located to avoid prior channels, because space available for sediment to fill may be diminished. To reach areas with sediment accommodation space, larger diversions are required periodically; this means that a suboptimal diversion must occasionally be engineered. However, we expect that the cost of a larger diversion will be assumed by the benefit provided under multiple shorter diversions.

We emphasize that there are real sociological, ecological, and practical challenges associated with artificial diversions (21, 25, 29), which we have grouped into a few parameters for simplicity. Grouping these factors into parameters gives incomplete attention to the human aspect of river diversions. Our formulation for societal benefit assumes that either 1) individual stakeholders will act collectively or 2) government will intervene on behalf of the common good, either of which increases total benefit accumulated over the delta land surface. However, river management has historically had an outsized negative impact on poor and minority communities, e.g., along the Mississippi River and Tennessee Valley (46–49). Stated explicitly, adverse diversion effects cannot be allowed to disproportionately impact disadvantaged communities, and our total benefit formulation does not adequately consider equity in determining optimal diversion placement (29). Thus, it remains a critically important open challenge to consider historical discrimination and disparate stakeholder interests in river management frameworks (29, 50, 51).

## Conclusion

Autogenic delta system behavior immediately following artificial diversions ${L_{D}}^{*}⪆0.5$ erodes the channel bed at the natural avulsion location, leading to a nonlinear scaling between diversion length and time to subsequent avulsion. Coupling this geomorphic constraint with a societal benefit assessment indicates that there is an optimal diversion length that minimizes flooding costs while maximizing societal benefit. Furthermore, the net benefit from an optimal diversion increases with flooding costs, which represents increased delta urbanization. Taken together, this indicates that urbanization increases the viability of sustainable deltaic diversion management plans, bolstering a promising outlook for these delicate landscapes.

## Materials and Methods

The numerical delta model (30) repeatedly computes one-dimensional gradually varying flow and sediment transport, coupled with a mass conservation equation, to simulate topographic change over an avulsion cycle (*SI Appendix*, section A). Deltaic lobe progradation and water discharge variability drive topographic change that creates channel superelevation and ultimately locates avulsions (30, 34). The one-dimensional channel is joined with a quasi–two-dimensional axis-symmetric delta topset and foreset to approximate delta evolution over multiple avulsion cycles. The delta model was run for several natural avulsion cycles and halted just before another avulsion, establishing the initial conditions for diversion modeling, which approximate a delta primed for avulsion (i.e., a diversion is imminently required to prevent an avulsion). Simulations represented a diversion by instantaneously modifying the channel bed from this state to one reflecting a new channel course from the diversion location to the sea (Fig. 1*C*).

The societal benefit framework is formulated to identify the optimal balance between river diversion cost and civil disruption by flooding. Focus is given to the subaerial delta landscape, and societal wellbeing is cast in terms of societal productivity and liabilities, integrated over the delta area and over one avulsion cycle. A complete derivation and description of assumptions are included in *SI Appendix*, section B. Parameter distributions were determined by data and estimates compiled from previous work (*SI Appendix*, section C).

## Supplementary Material

Supplementary File

## Data Availability

Code and model results data have been deposited in GitHub, https://github.com/amoodie/paper_resources/tree/master/Moodie_deltasustainability.
