# Supplementary material for: Optimized river diversion scenarios promote sustainability of urbanized deltas
Source: Proc Natl Acad Sci U S A. 2021 Jun 28;118(27):e2101649118. doi: 10.1073/pnas.2101649118 (PMC8271632; doi:10.1073/pnas.2101649118)
Supplement: Supplementary File [file pnas.2101649118.sapp.pdf]

Supplementary Information for: Optimized river diversion scenarios promote sustainability of urbanized deltas

Andrew J. Moodie,[1,2*] Jeffrey A. Nittrouer[1,3]

[1]Department of Earth, Environmental and Planetary Sciences, Rice University
Houston, Texas, 77005 USA
[2]Department of Civil, Architectural and Environmental Engineering, University of Texas at Austin
Austin, Texas, 78712 USA
[3]Present address Department of Geosciences, Texas Tech University
Lubbock, Texas 79409 USA
*Corresponding author: amoodie@utexas.edu

A Delta model development and simulating artificial diversions

A.1 Numerical model of Moodie et al., 2019

We used the numerical delta model of Moodie et al., 2019 [1], which simulates delta evolution over multiple avulsion cycles. The numerical model computes one-dimensional gradually-varying flow via a backwater equation:

$$\frac{dH}{dx} = \frac{S - C_f \mathrm{Fr}^2}{(1 - \mathrm{Fr}^2)} + \frac{\mathrm{Fr}^2}{(1 - \mathrm{Fr}^2)} \frac{H}{B} \frac{dB}{dx}, \tag{S1}$$

where H is the flow depth, x is a downstream directed coordinate, slope $S = d\eta/dx$ for the bed elevation η, $C_f = 0.001$ is the dimensionless coefficient of friction for the Yellow River [2], $\mathrm{Fr}^2 = Q_w^2/gB^2H^3$ is the Froude number for a rectangular channel, g is the gravitational acceleration constant, and B is the width of the flow.

Sediment transport per-unit-flow width (q_s) is computed for the Yellow River system as:

$$q_s = \sqrt{RgD_{50}^3} \frac{\alpha}{C_f} \tau_*^n, \tag{S2}$$

where R is the submerged specific gravity of sediment, $D_{50} = 90\ \mu m$ is the median grain diameter of the bed-material [2, 3], $\tau_* = C_f U^2/RgD_{50}$ is the Shields number, and $\alpha = 0.895$ and $n = 1.678$ are adjusted coefficients to the generalized form of the [4] equation fit for the lower Yellow River [2, 5].

Sediment mass conservation maintains the change in bed elevation over time:

$$(1 - \phi) \frac{\partial \eta}{\partial t} = -\frac{\partial Q_s}{\partial x} \frac{1}{B_e}, \tag{S3}$$

where t is time, $\phi = 0.4$ is the channel-bed porosity, $Q_s = q_s B_c$ is the sediment flux over the flow width, and B_e is the effective width of sediment deposition [6], defined by a piecewise function representing the

Table S1: Parameterization for spin up and artificial diversion simulations. Additional parameters set to values defined in [1].

Parameter	Symbol	Model input	Units
porosity	ϕ	0.4	1
domain length	L	400	km
spatial step	dx	0.66	km
time step	dt	8–21600	s
median grain size	D_{50}	90	μm
initial bed slope	S_0	6.4×10^{-5}	1
bankfull discharge	Q_{bf}	3000	m^3/s
bankfull flow depth	H_{bf}	4.5	m
hydrograph		artificial	
backwater length-scale	L_b	40	km
lobe width	B_o	9	km

combined widths of the channel (B_c), floodplain (B_f), and/or delta-lobe (B_o) [1]:

$$B_e(x) = \begin{cases} B_c + B_f & : x \leq r \\ B_c + B_o & : r < x \leq m \\ B_c & : x > m \end{cases}, \tag{S4}$$

where r denotes the edge of the delta topset and m is the mouth position at the end of the lobe.

During the model spin-up simulations, avulsions are allowed to occur naturally; no avulsions occur during diversion simulations. Avulsion occurs when sediment aggradation on the channel bed elevates the bankfull water surface ($\eta + H_{bf}$) to a critical height above the surrounding floodplain (i.e., superelevation) [1, 8, 9]. Superelevation (ΔZ) is defined as:

$$\Delta Z(x) = \eta(x) + H_{bf} - Z(x), \tag{S5}$$

where Z is the delta topset elevation (determined by long-term delta evolution routine described below and in [1]). So, the necessary condition for avulsion during the model spin up is:

$$\Delta Z > \beta H_{bf}, \tag{S6}$$

where β is a coefficient set to vary between $\beta = 0.4$–0.5, thus scaling avulsions $L_A \approx L_b$ and deltaic lobe length $L_L \approx 0.5 L_b$ [1]. If this condition is satisfied during the model spin-up, then an avulsion is triggered at the appropriate spatial x-coordinate at time t.

The one-dimensional hydraulic model is coupled with a two-dimensional axisymmetric delta system with protruding lobe (Figure S1). To represent avulsions occurring over multiple avulsion cycles in the model spin up, sediment mass is redistributed in a axisymmetric 2D delta framework [1]. When an avulsion is triggered, the volume of sediment deposited within the lobe portion of the model domain is distributed along the entire delta coastline, thus prograding the delta. Simultaneously, the delta system topset is aggraded by the sediment deposited within the model floodplain at each x-coordinate. Both of these sediment redistributions occur according to mass conservation [1]. This method of redistributing lobe and floodplain sediment averages deltaic processes occurring over multiple lobe progradation and avulsion cycles.

A selection of parameters used in the model spin-up and diversion simulations are listed in Table S1; additional model parameters are set to values defined in [1].

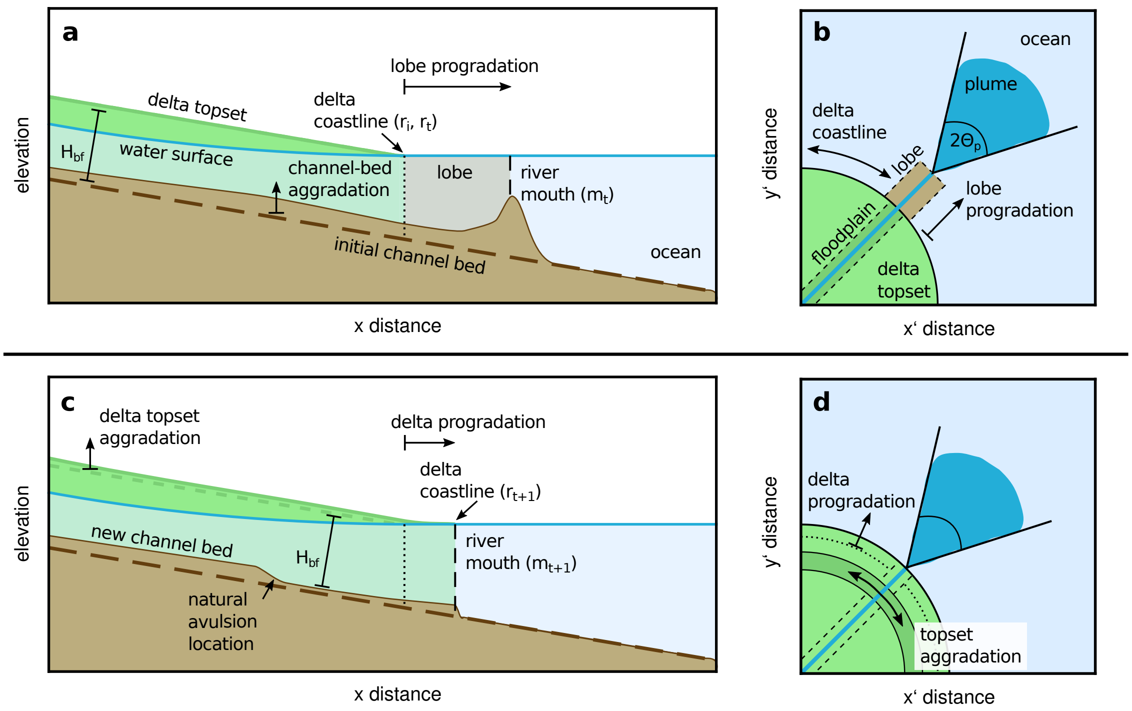

Figure S1: Schematic (not drawn to scale) depicting numerical model immediately prior to a natural avulsion in a) the one-dimensional long profile, showing the subaerial delta topset, change in channel bed elevation (shaded brown area) from the initial channel bed a bankfull flow depth below the topset (thick dark-brown line), water surface (blue line), the initial delta coastline position (which is also the initial mouth location before lobe progradation), and the current river mouth position and extent of lobe (shaded brown). b) Planform depiction of the delta system for the same time as (a) (the long profile would be a slice down the 45° axis), the floodplain (shaded in dark green) and a developed lobe (shaded in brown) depict the model depositional area. Θ_p is the offshore-plume spreading angle, here set to 5° after [7]. c) Long profile and d) planform schematic depicting numerical model immediately following a natural avulsion. Sediment in the delta lobe is redistributed along the delta front, and sediment deposited in the floodplain is redistributed axisymmetrically across the delta topset over the annulus area for each x-coordinate, thereby prograding and aggrading the delta. The channel bed is linearly interpolated to a bankfull flow depth below the topset for locations downstream of the avulsion location. See Main Text Figure 1 for a depiction of the channel adjustment following an artificial diversion. Modified with permission from SI ref. 1.

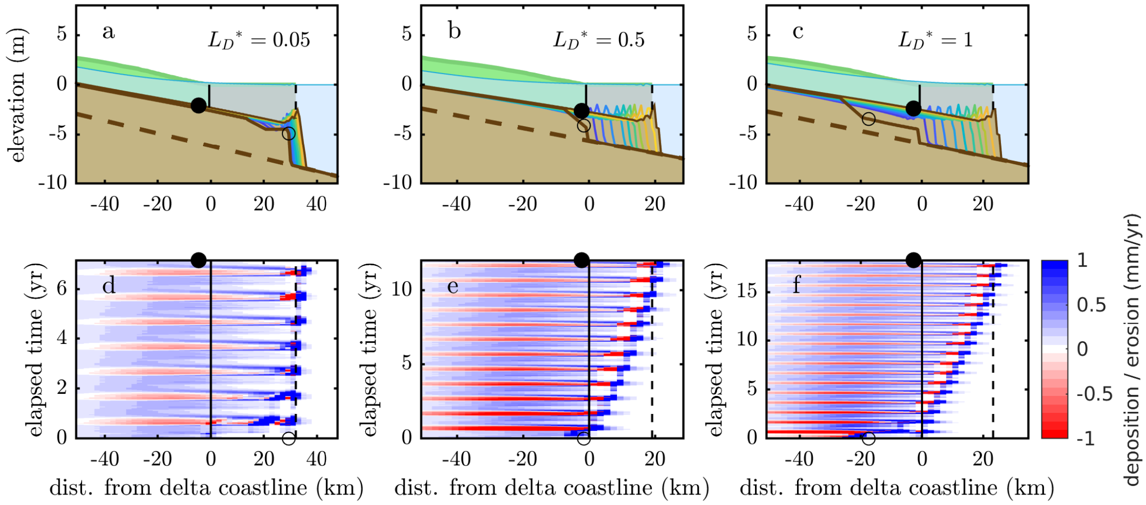

Figure S2: Long profile of model simulations for artificial diversions at a) $L_D{}^* = 0.05$, b) $L_D{}^* = 0.5$, and c) $L_D{}^* = 1.0$. Open circles mark the location of artificial diversions, and closed circle is where cycle-ending superelevation conditions were reached. Vertical lines mark the delta coastline (solid) and river mouth at T_A (dashed) [1]. Spatiotemporal pattern of erosion and deposition during each simulation for d) $L_D{}^* = 0.05$, e) $L_D{}^* = 0.5$, and f) $L_D{}^* = 1.0$. Marker symbols and lines are same as in panels a–c.

A.2 Artificial diversion channel bed evolution

Artificial diversion simulations were set up by evolving the numerical model (Appendix Section A.1) over 14–15 avulsion cycles, using parameters and a hydrograph outlined in [1] and Table S1. Of particular note, we varied the threshold superelevation value $\beta = 0.4$–0.5 across the simulations, which scales avulsions $L_A{}^* \approx 1$ and deltaic lobes $L_L \approx 0.5 L_b$.

We then simulated artificial diversions from $L_D{}^* = 0.05$–1.6, whereby a channel pathway is cut to one bankfull flow depth below the delta topset [8], from the diversion site to the coastline (L_f, 1b, Appendix Section A.1). For diversions on the delta lobe, the coastline is defined as the location R_l distance from the diversion site. The diversion site is simulated as a constant-slope reach of the channel bed that connects the upstream channel bed with the newly-cut channel bed [1]. The simulation is run until channel superelevation is reached that would lead to a natural avulsion at some distance upstream from the channel mouth (L_A).

We conducted ten simulations at each diversion length (Figure 2). Using different set-up cycles and superelevation thresholds for our simulations allowed us to assess the variability and sensitivity of the relationship between $L_D{}^*$ and $T_A{}^*$.

Artificial diversion simulations for the Yellow River delta display complex spatiotemporal behavior. In this section, we describe three characteristic simulations in detail ($L_D{}^* \in \{0.15, 0.5, 1.0\}$). Figure S2a–c shows the time evolution of the model long-profile over the avulsion cycle following the artificial diversion. The thick brown line depicts the channel bed immediately following the artificial diversion, and the colored lines → the thin brown line depict the bed configuration through time and at the final state, respectively [1].

Following artificial diversion at $L_D{}^* = 0.05$, the channel bed was reworked at the diversion location, but the bed elevation was only minimally lowered at the diversion location or the natural avulsion location (Figure S2a). A mouth bar was rapidly deposited (Figure S2a). During each year of the simulation,

channel bed erosion occurred along most of the delta, leading to sediment deposited in the channel mouth bar (Figure S2d). After 2.7 yr, channel superelevation conditions were reached and the simulation was ended.

Artificial diversion at ${L_D}^* = 0.5$ led to an increase in time to next avulsion of ∼11 yr (Figure S2b). In this simulation, the channel bed was substantially reworked at the diversion location and as a result, the bed was lowered at the natural avulsion location (${L_A}^*$, Figure S2b). Sequences of flood discharge each year eroded the channel bed upstream and deposited this sediment in a prograding delta lobe and mouth bar (Figure S2e).

Artificial diversion at ${L_D}^* = 1.0$ led to substantial channel bed reworking at, and upstream of, the diversion location (Figure S2c). Time to next avulsion was ∼18 yr. In this simulation, the channel bed was artificially lowered at the natural avulsion location (${L_A}^*$), but autogenic reworking lowered the bed farther (Figure S2c). Sequences of flood discharge each year eroded the channel bed upstream and deposited this sediment in a prograding delta lobe and mouth bar (Figure S2f).

B Societal benefit formulation

B.1 Complete derivation

Levee-breaching floods occur on a delta system at a frequency proportional to the expected time between natural avulsions (T_A, the avulsion timescale), and cover an area a_f. Assuming a flooding damage rate C_f per unit area per unit time ($\$/\mathrm{L}^2{\cdot}\mathrm{T}$), the expected cost of flooding per unit time is $a_f C_f$. Land area that is not flooded produces revenue at a rate C_p per unit area per unit time ($\$/\mathrm{L}^2{\cdot}\mathrm{T}$).

During the time between avulsions, the delta system progrades a lobe, building land area (a_l) with fixed width (B_l) and length that increases with time $L_l = r_l t$, where r_l is a progradation rate, and t is time:

$$a_l = L_l B_l = r_l t B_l. \tag{S7}$$

Geometric scaling, physical experiments, and numerical modeling suggest that lobe length at the time of avulsion is proportional to channel bed superelevation and the backwater length $L_l \approx r_l T_A \approx \beta L_b$ [1, 9, 10]. Lobe revenue is given by $a_l C_l$, where C_l is the lobe land revenue per unit area per unit time.

Avulsions may be natural or artificially created, incurring indirect costs associated with land flooding. Natural avulsions have no direct cost, but the cost of an artificial diversion is composed of a fixed cost ($C_{D,x}$) and a variable cost set by the diversion length ($C_{D,v}$):

$$C_D = C_{D,x} + L_f C_{D,v}. \tag{S8}$$

We assume that $C_{D,v}$ incorporates costs associated with constructing a new channel including acquiring land, trenching, and levee breaching.

The benefit (Π) of an anthropogenically managed delta over an avulsion cycle (T_A) is:

$$\Pi = C_p(a_d - a_f)T_A - C_D + \int^{T_A} r_l t B_l C_l + C_p a_f(1 - I(t)) - C_f a_f I(t)\ dt, \tag{S9}$$

where a_d is the total delta area, $I(t)$ is an indicator function with value 1 when flooding occurs during t, and 0 when there is no flooding during t. We assume that there is only one flooding event per avulsion cycle T_A, which makes the integral $\int^{T_A} I(t)\ dt = 1$ unit time ($\equiv T_A(1/T_A)$). The integral for the lobe is simply

$$\pi_l = \int^{T_A} r_l t B_l C_l\ dt \tag{S10}$$

$$\pi_l = \frac{1}{2} r_l B_l C_l {T_A}^2 \tag{S11}$$

which is recast given the scaling above ($L_l \approx r_l T_A \approx \beta L_b$):

$$\pi_l = \frac{1}{2}\beta L_b B_l C_l T_A \tag{S12}$$

is the benefit from the lobe over the avulsion cycle T_A. Evaluating the integral over T_A gives:

$$\Pi = C_p a_d T_A - C_p a_f T_A - C_{D,x} - L_f C_{D,v} + \frac{1}{2}\beta L_b B_l C_l T_A + C_p a_f T_A \Big(1 - \frac{1}{T_A}\Big) - C_f a_f, \tag{S13}$$

We nondimensionalize the parameters in Equation S13 with the linear transformations of the backwater length (L_b), the long-term average avulsion timescale ($T_{A,0}$), and a characteristic cost (C_c):

$$a_d{}^* = a_d/[L_b{}^2] \tag{S14}$$
$$a_f{}^* = a_f/[L_b{}^2] \tag{S15}$$
$$C_p{}^* = C_p/[C_c] \tag{S16}$$
$$C_f{}^* = C_f/[C_c] \tag{S17}$$
$$C_l{}^* = C_l/[C_c] \tag{S18}$$
$$C_{D,x}{}^* = C_{D,x}/[C_c L_b{}^2] \tag{S19}$$
$$C_{D,v}{}^* = C_{D,v}/[C_c L_b] \tag{S20}$$
$$\Pi^* = \Pi/[C_c L_b{}^2] \tag{S21}$$
$$L_A{}^* = L_A/L_b \tag{S22}$$
$$L_f{}^* = L_f/L_b \tag{S23}$$
$$L_l{}^* = L_l/L_b \tag{S24}$$
$$T_A{}^* = T_A/T_{A,0} \tag{S25}$$

Applying Equations S14–S25 to Equation S13 gives:

$$\begin{aligned}\Pi^* C_c L_b{}^2 = C_p{}^* C_c a_d{}^* L_b{}^2 T_A{}^* T_{A,0} - C_p{}^* C_c a_f{}^* L_b{}^2 T_A{}^* T_{A,0} - \\ C_{D,x}{}^* C_c L_b{}^2 - L_f{}^* L_b C_{D,v}{}^* C_c L_b + \frac{1}{2}\beta L_b B_l C_l{}^* C_c T_A{}^* T_{A,0} + \\ C_p{}^* C_c a_f{}^* L_b{}^2 T_A{}^* T_{A,0} - C_p{}^* C_c a_f{}^* L_b{}^2 - C_f{}^* C_c a_f{}^* L_b{}^2.\end{aligned} \tag{S26}$$

Equation S26 is simplified by dividing all terms by the coefficient on the highest order term, the delta area ($C_c L_b{}^2 C_p{}^*$):

$$\begin{aligned}\frac{\Pi^*}{C_p{}^*} = a_d{}^* T_A{}^* T_{A,0} - a_f{}^* T_A{}^* T_{A,0} - \frac{C_{D,x}{}^*}{C_p{}^*} - L_f{}^* \frac{C_{D,v}{}^*}{C_p{}^*} \\ + \frac{1}{2}\beta \frac{B_l}{L_b}\frac{C_l{}^*}{C_p{}^*} T_A{}^* T_{A,0} + a_f{}^* T_A{}^* T_{A,0} - a_f{}^* - \frac{C_f{}^*}{C_p{}^*} a_f{}^*.\end{aligned} \tag{S27}$$

Equation S27 is simplified by eliminating coefficients. Setting the coefficients of the first term equal to unity and solving for the characteristic timescale:

$$a_d{}^* T_{A,0} = 1 \quad \rightarrow \quad T_{A,0} = \frac{1}{a_d{}^*}. \tag{S28}$$

Substituting Equation S28 into Equation S27 and simplifying gives:

$$\frac{\Pi^*}{C_p{}^*} = T_A{}^* - \frac{C_{D,x}{}^*}{C_p{}^*} - L_f{}^* \frac{C_{D,v}{}^*}{C_p{}^*} + \frac{1}{2}\beta \frac{B_l}{L_b}\frac{C_l{}^*}{C_p{}^*}\frac{1}{a_d{}^*} T_A{}^* - a_f{}^* - \frac{C_f{}^*}{C_p{}^*} a_f{}^*. \tag{S29}$$

To reduce the number of cost variables, we assume that artificial diversion costs are related through a fixed ratio α, such that ${C_{D,v}}^* = \alpha {C_{D,x}}^*$, and define substitutive equations as:

$$\lambda_\Pi = \frac{\Pi^*}{{C_p}^*}, \quad \lambda_f = \frac{{C_f}^*}{{C_p}^*}, \quad \lambda_l = \frac{{C_l}^*}{{C_p}^*}, \quad \lambda_D = \frac{{C_{D,x}}^*}{{C_p}^*}, \quad R_l = \frac{B_l}{L_b}. \tag{S30}$$

Applying these assertions to Equation S29 gives:

$$\lambda_\Pi = {T_A}^* - \lambda_D - \alpha {L_f}^* \lambda_D + \frac{1}{2}\beta R_l \lambda_l \frac{1}{{a_d}^*}{T_A}^* - {a_f}^* - \lambda_f {a_f}^*, \tag{S31}$$

and after consolidating terms:

$$\lambda_\Pi = {T_A}^*\Big[1 + \frac{1}{2}\beta R_l \lambda_l \frac{1}{{a_d}^*}\Big] - \lambda_D\Big[1 + \alpha {L_f}^*\Big] - {a_f}^*\Big[1 + \lambda_f\Big]. \tag{S32}$$

Finally, we are interested in benefit change per unit dimensionless time:

$$\frac{\lambda_\Pi}{{T_A}^*} = 1 + \frac{1}{2}\beta R_l \lambda_l \frac{1}{{a_d}^*} - \frac{\lambda_D}{{T_A}^*}\Big[1 + \alpha {L_f}^*\Big] - \frac{{a_f}^*}{{T_A}^*}\Big[1 + \lambda_f\Big]. \tag{S33}$$

Radially symmetric delta area is [1]:

$$a_d = \frac{\pi}{4}{L_d}^2 \quad \therefore \quad {a_d}^* = \frac{\pi}{4}{L_d}^{*2}, \tag{S34}$$

where $\pi \approx 3.1415$. The flooded area from a levee breaching flow (a_f) on an axisymmetric delta [1] is approximately:

$$a_f \approx \tan\frac{\theta}{2}{L_f}^2, \tag{S35}$$

where θ is an opening angle of flooded area from the levee breach, and $L_f = L_D - L_l$ is the distance from levee breach to the radially-averaged shoreline for avulsions upstream of the delta lobe, and $L_f = B_l/2$ for avulsions occurring on the delta lobe. This approximation for flooded area is validated in Appendix Section B.2.

Nondimensionalized flooding area is a piecewise function:

$${L_f}^* = \begin{cases} R_l/2 & : {L_D}^* \leq \beta \\ {L_D}^* - {L_l}^* \equiv {L_D}^* - \beta & : {L_D}^* > \beta \end{cases}. \tag{S36}$$

Finally, Equation S33 becomes:

$$\frac{\lambda_\Pi}{{T_A}^*} = 1 + \frac{1}{2}\beta R_l \lambda_l \frac{1}{\frac{\pi}{4}{L_d}^{*2}} - \frac{\lambda_D}{{T_A}^*}\Big[1 + \alpha {L_f}^*\Big] - \frac{\tan\frac{\theta}{2}{L_f}^{*2}}{{T_A}^*}\Big[1 + \lambda_f\Big]. \tag{S37}$$

Equation S37 depends on seven dimensionless parameters λ_f, λ_l, λ_D, $\tan(\theta/2)$, β, α, and R_l. There are three variables in Equation S37 (${L_f}^*$, ${T_A}^*$, and ${L_d}^*$). In this text, we fix ${L_d}^* = 1$, and cast ${L_f}^*$ and ${T_A}^*$ in terms of ${L_D}^*$ (Equation S36 and relationships in main text), reducing the number of variables of interest to one (${L_D}^*$):

$$\frac{\lambda_\Pi}{{T_A}^*} = 1 + \frac{2}{\pi}\beta R_l \lambda_l - \frac{\lambda_D}{{T_A}^*}\Big[1 + \alpha {L_f}^*\Big] - \tan(\theta/2){L_f}^{*2}\frac{1}{{T_A}^*}\Big[1 + \lambda_f\Big]. \tag{S38}$$

For clarity in the main text, we define this equation as $\lambda_{\Pi,\text{diversion}}$ (Equation 3, main text).

In this societal benefit formulation, we ignore the time value of money. We make this choice because the avulsion timescale on many delta systems is 10^2–10^3 yr, and it would be speculative to apply any

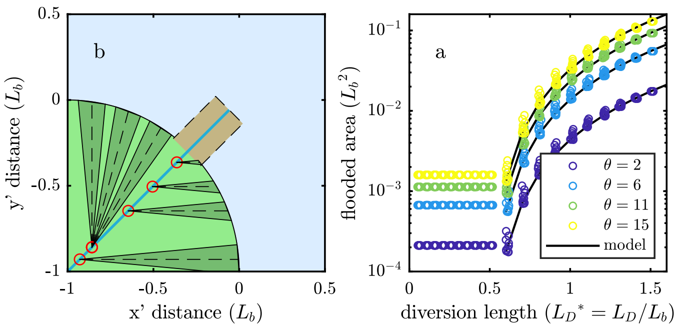

Figure S3: a) Radially symmetric delta framework evaluations of flooded area, demonstrating pathways of flooding for artificial diversions at any length along the channel course (lower half) and along various trajectories to the shoreline. b) Evaluations and model prediction for flooded area as a function of θ and $L_D{}^*$.

predicted discount rate over these timescales. Additionally, we make the simplifying assumption that economic parameters are fixed over an avulsion cycle; that is, λ parameters do not change over T_A. This assumption makes sense when $T_A = 10^0$ yr, but is likely unrealistic when $T_A = 10^3$ yr. Nevertheless, a representative value for λ parameters can be selected that is expected to reflect the societal interests over the coming avulsion cycle.

Another assumption was to ignore that surface flooding likely becomes more common as channel superelevation increases prior to avulsion [9, 11]. We assume these floods are contained by levee engineering, and are an insignificant cost compared to levee-breaching floods. Our assumption may be true where floodplain use is limited to agriculture, but would be erroneous if significant infrastructure exists on the floodplain. In the future, non-levee-breaching flood costs could be incorporated via another term with a probability distribution for flood damage that varies over the avulsion cycle.

B.2 Approximating function for flooded area

The approximation used for flooded surface area a_f (Equation S35) is a trigonometric derivation. Let the area flooded be equal to a triangle with opening angle θ and height $h = L_f$. The triangle thus has base $b/h = 2\tan(\theta/2)$. Substituting this equation into the area for a triangle $a = (1/2)bh$, and simplifying, gives Equation S35.

We validated this simplified formulation for flooded area with calculations of the area in our radially symmetric delta framework. In this framework, we can evaluate artificial diversions at any length along the channel course (lower half, Figure S3a), and direct the new channel course along any trajectory to the shoreline (upper half, Figure S3a). We computed the area using Heron's Formula, where $A = \sqrt{s(s-a)(s-b)(s-c)}$ and $s = (a+b+c)/2$ is triangle semi-perimeter.

We found that the simple representation works extremely well, capturing the systematic variability in flooded area due to change in θ and $L_D{}^*$ (Figure S3b). Additional variability that is not explained by the model is due to the angle the flood is directed at the shoreline. Levee-breaching floods directed at a high angle to the channel traverse a longer distance to the shoreline, and cover larger flood areas (Figure S3b). The model is thus a conservative estimate for flooded area due to levee breaching.

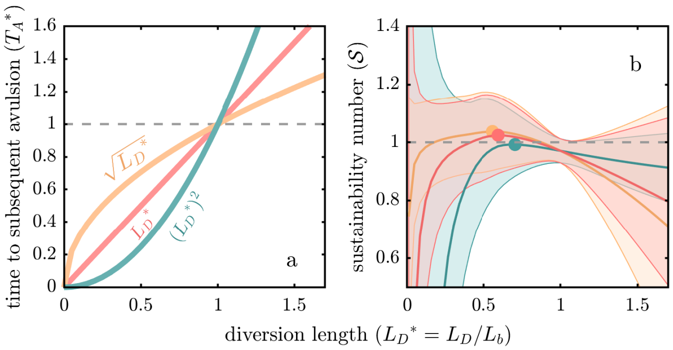

Figure S4: a) Three functions relating diversion length ($L_D{}^*$) to time to subsequent avulsion ($T_A{}^*$) described in the text as Equations S39–S41. b) resulting evaluations of the sustainability number for each function; shaded region is the 95% confidence envelope for results.

B.3 Parameter space of societal benefit functions

B.3.1 Alternative functions relating $L_D{}^*$ to $T_A{}^*$

Engineered diversions generate a new channel on the floodplain, the volume of which is proportional to diversion length (L_D) and channel depth and width [12, 13]. Once equilibrium depth and width are established, this channel is prone to sedimentation due to backwater hydrodynamics; hence, diversion length influences sedimentation and the propensity for avulsion setup. For simplicity, if we assume spatially uniform deposition, a null hypothesis for time to subsequent avulsion is that it scales linearly with diversion length: $T_A{}^* = L_D{}^*$, where the relation is cast in dimensionless time ($T_A{}^* = T_A/T_{A,0}$) and length ($L_D{}^* = L_D/L_b$). Alternatively, a diversion upstream of the backwater segment bypasses any portion of the previous channel that was subjected to enhanced aggradation prior to the diversion; in such a case, the time to a subsequent avulsion may increase nonlinearly with diversion length.

In the main text, we define a single function to relate the time to subsequent avulsion with the diversion length:

$$T_A{}^* = \sqrt{L_D{}^*}. \tag{S39}$$

This function arises from an understanding of nonlinear backwater sedimentation effects, and is anchored such that the time to subsequent avulsion is equal to the long-term average avulsion timescale, if a diversion is located at the natural location ($T_A{}^* = 1$ when $L_D{}^* = 1$). In the analyses of the model simulations in the main text, we determine the simulation results parallel Equation S39.

However, the form of Equation S39 is chosen for simplicity, was not fit to model results, and likely varies between delta systems. In an effort to demonstrate that our societal benefit formulation leads to an optimum regardless of the relationship between time to subsequent avulsion and diversion length (i.e., our findings are robust), we define two alternative relationships and evaluate the sustainability number for these functions. The two alternative functions we explore (which are also consistent with expected anchor points at $L_D{}^* = 0$ and $L_D{}^* = 1$) are:

$$T_A{}^* = (L_D{}^*) \tag{S40}$$

$$T_A{}^* = (L_D{}^*)^2. \tag{S41}$$

Figure S4a shows the form of Equations S39–S41, and Figure S4b shows the resulting evaluations of the sustainability number for each function from a Monte Carlo simulation using parameter distributions described in the main text. For all functions, an optimum exists for $L_D{}^* = 0.5$–0.7.

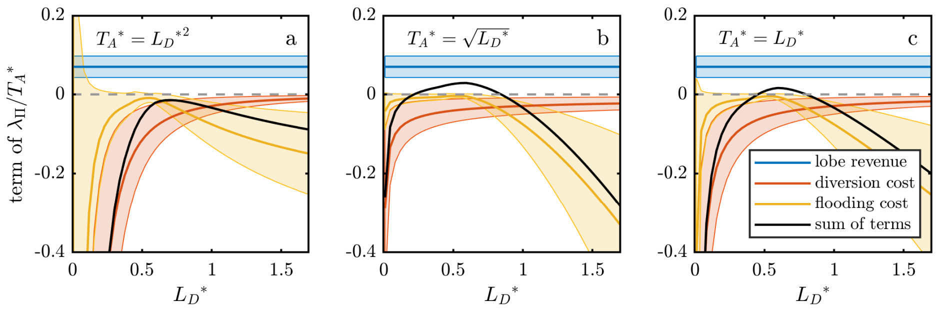

Figure S5: Components of artificial diversion benefit formulation (Equation 3) for three theoretical relationships between $T_A{}^*$ and $L_D{}^*$. The form of each component is similar in each relationship, but the sum of components depends on the interaction of each curve, leading to various optimal diversion locations for each relationship.

B.3.2 Components of artificial diversion function

We evaluated each term of the societal benefit equation individually (Equation 3, S38), to assess which terms contribute to nonlinearity and optimality of the sustainability number (Figure S5). We excluded the delta area constant (i.e., 1) from this analysis, because it does not depend on any parameters or variables.

The first term ($\frac{2}{\pi}\beta R_l \lambda_l$), which we call the "lobe revenue" term, depends only on physical delta parameters and lobe revenue parameter λ_l. Thus, for stationary Monte Carlo sampling distributions, the value of the lobe revenue term is constant for all $L_D{}^*$ and equal to $\frac{2}{\pi}\mathbb{E}\left[\beta R_l \lambda_l\right]$ (Figure S5).

The second term ($-\frac{\lambda_D}{T_A{}^*}\left[1+\alpha L_f{}^*\right]$), which we call the "diversion cost" term, depends on λ_D and $L_D{}^*$ through formulations for $T_A{}^*$ and $L_f{}^*$. The diversion cost term always tends towards zero, regardless of the relationship between $T_A{}^*$ (Figure S5), though the asymptote depends on this relationship. Overall, this is to say that the impact of the diversion term on the sustainability number is diminished with increasing $L_D{}^*$.

The third term ($-\frac{[1+\lambda_f]}{T_A{}^*}\tan(\theta/2){L_f{}^*}^2$), which we call the "flooding cost" term, depends on λ_f and $L_D{}^*$. This cost initially decreases with increasing $L_D{}^*$, because $L_f{}^*$ is fixed while $L_D{}^* \leq \beta$, whereas $T_A{}^*$ increases over the same interval. For diversions $L_D{}^* > \beta$ the flooding cost increases exponentially.

For all theoretical relationships linking $T_A{}^*$ and $L_D{}^*$, there is an optimal dimensionless societal benefit per unit time ($\lambda_f/T_A{}^*$, Figure S5). The optimum arises due to the competition of increasing time to next avulsion ($T_A{}^*$) and flooded area ($a_f{}^*$) as diversion length ($L_D{}^*$) increases. The location of the optimum depends on how quickly $T_A{}^*$ increases, because the diversion costs (λ_D) are amortized over this time.

B.3.3 Components of sustainability number function

The sustainability number contrasts the societal benefit derived from an artificial diversion to the benefit derived from allowing natural avulsion (Equation 5). This section examines the components of the sustainability number evaluations that varied the flooding cost parameter λ_f (Figure 4d).

The societal benefit from a natural delta management strategy does not depend on diversion length (Figure S6, dashed lines). However, the value of the benefit decreases strongly with increasing flooding costs (Figure S6), such that ∼25% of value is eroded for a 10x increase in flooding cost. The benefit of

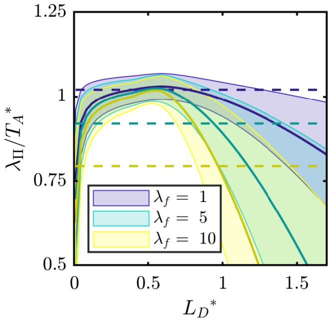

Figure S6: Components of sustainability number (Equation 5) as a function of increasing flooding cost parameter (λ_f). Dashed line is the benefit derived from natural delta management (i.e., the denominator in Equation 5) and the solid line with shading is the mean and 95% confidence interval for the benefit from artificial diversions (i.e., the numerator in Equation 5). Data correspond to Figure 4d.

artificial diversion strategies is minimally impacted by changing flooding costs, for diversions ${L_D}^* \lessapprox 0.7$ (Figure S6), but benefit decreases faster with larger diversions ${L_D}^* > 0.7$.

Taken together, the disparity between the natural avulsion strategy and diversions increases with flooding cost, driving up the sustainability number (Figure 4d). The sustainability number is always less than unity for diversions at ${L_D}^* = 1$. This is necessarily so, because this length scale equates to *paying* for the same outcome as allowing natural diversion. This is due to our assumption that natural diversions occur at ${L_D}^* = 1$; this assumption is based on long-term average behavior of delta systems and should not be expected to be true for any single avulsion cycle. Thus, our framework neglects an important benefit gained from artificial diversions: certainty.

B.3.4 Effect of parameter change on sustainability number

We examined the sensitivity of the sustainability number (Equation 5) to the range of each dimensionless parameter (λ_D, λ_f, λ_l, α, β, θ, and R_l) versus the artificial diversion length (${L_D}^*$). For this analysis, we used the relation ${T_A}^* = \sqrt{{L_D}^*}$ (Equation S39) as a predictor of the time to subsequent avulsion for a given diversion length. The results are shown in Figure S7. We found that all parameter spaces have an optimal artificial diversion length.

C Constraining dimensionless parameters

C.1 Levee-breach angle

We measured levee-breaching flows in the Tulane Delta Basin 12-1 experiment (`http://sedexp.net/catalog/tdb121-tulane-delta-basin`). We measured angles using the Levee-breach Angle Finder tool (`https://github.com/amoodie/levee-breach_anglefinder`), but hid the angle measured during data collection to avoid introducing measurement bias.

Levee breaches were identified based on the presence of surface water in overhead images from the delta experiment. We used a combination of 1) color shade to indicate flow depth and 2) presence of darker sediment to indicate coarse sediment suspension, to identify levee breaches in the dataset. Usually, flow exited the channel along a ∼10 cm long section of the levee, and spread over the delta surface before reaching the shoreline. Thus, our approach was to call the centerpoint of the levee breach the breach

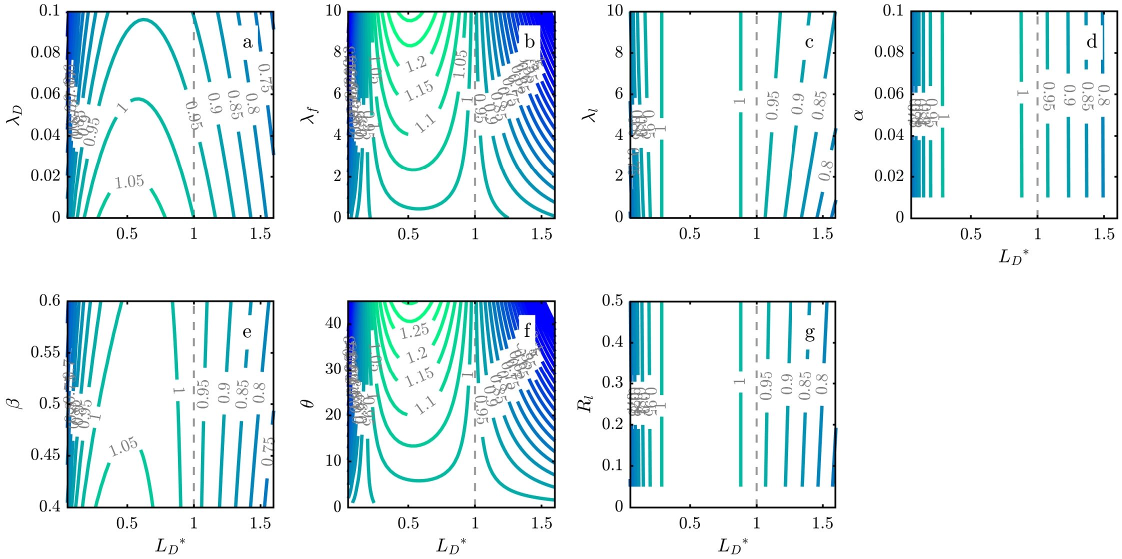

Figure S7: Contours of sustainability number (Equation 5) for range of dimensionless parameters a) λ_D, b) λ_f, c) λ_l, d) α, e) β, f) θ, and g) R_l, versus diversion length ($L_D{}^*$). An optimal diversion location persists for all parameter spaces.

apex, and identify the points of intersection between the flow edges and the delta shoreline as endpoints of the surface flow.

We were careful to avoid crevassing associated directly with avulsion and channel sweeping, because these events usually involved many small breaches that persisted in the levee. In these breaches, flow coalesced on the delta topset, which made it impossible to attribute surface flooding to any point along the channel. We also avoided crevassing near the walls of the experiment, because flooding could not naturally spread there.

The identified levee breaches ($n = 60$) follow a gamma distribution, which we identified as having shape parameter $a = 2.0$, scale parameter $b = 6.0$, and location parameter $\mu = 7$; this equates to an expected value $\mathbb{E}[\theta] = 12°$. Additionally, we measured the only levee-breaching avulsion on the Yellow River delta and found the breach angle $26 \pm 7°$, based on independent measurements by three people.

C.2 Dimensionless cost parameters

Data constraining cost parameters are limited in the literature. Values for λ_f and λ_l were largely based on the intuitive definition of these parameters. For example, the flooding cost parameter characterizes damage from flooding to land-use revenue, and thus equals unity when exactly an entire unit-time productivity has been eliminated by a unit-time flood.

We relied largely on data collated by [11] to determine appropriate ranges to explore with our societal benefit model. These authors suggest that artificial diversion cost on the Yellow River delta would be composed of a ¥180 MM fixed construction cost, and a ¥2 MM/km variable cost. The authors documented flooding damage costs on the Yellow River delta per unit area as approximately ¥6 MM/km^2, and equate this value to roughly one year's food crop.

We thus find the value of λ_f to be order unity (flood damage / food crop), and is probably greater than unity. Lobe value is also assumed to be order unity. The artificial diversion cost is scaled according

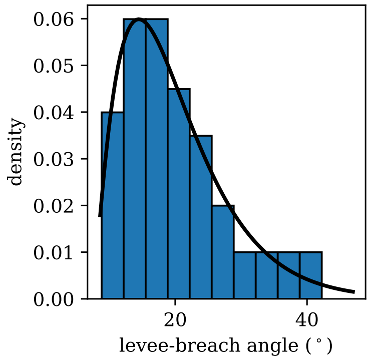

Figure S8: Histogram and fitted gamma distribution for measured levee-breach angles ($n = 60$).

to Equation S30 (i.e., $1/{L_b}^2$), and thus is $\lambda_D = 180/6/(40^2) \approx 0.02$ for the Yellow River delta.

References

[1] Andrew J. Moodie et al. "Modeling Deltaic Lobe-Building Cycles and Channel Avulsions for the Yellow River Delta, China". In: *Journal of Geophysical Research: Earth Surface* 124.11 (2019), pp. 2438–2462. DOI: `https://doi.org/10.1029/2019JF005220`.

[2] Hongbo Ma et al. "The exceptional sediment load of fine-grain dispersal systems: Example of the Yellow River, China". In: *Science Advances* 3 (2017), p. 7. DOI: `10.1126/sciadv.1603114`.

[3] Andrew J. Moodie et al. "Suspended-sediment induced stratification inferred from concentration and velocity profile measurements in the flooding lower Yellow River, China". In: *Water Resources Research* (2020). DOI: `10.1029/2020WR027192`.

[4] Frank Engelund and Eggert Hansen. *A monograph on sediment transport in alluvial streams*. Technisk Vorlag, Copenhagen, Denmark, 1967.

[5] Hongbo Ma et al. "Universal relation with regime transition for sediment transport in fine-grained rivers". In: *Proceedings of the National Academy of Sciences* (2020). DOI: `10.1073/pnas.1911225116`.

[6] Phairot Chatanantavet and Michael P. Lamb. "Sediment transport and topographic evolution of a coupled river and river plume system: An experimental and numerical study". In: *Journal of Geophysical Research: Earth Surface* 119.6 (June 2014), pp. 1263–1282. ISSN: 21699003. DOI: `10.1002/2013JF002810`. (Visited on 09/23/2016).

[7] Michael P. Lamb et al. "Backwater and river plume controls on scour upstream of river mouths: Implications for fluvio-deltaic morphodynamics". In: *Journal of Geophysical Research* 117.F1 (Jan. 2012). ISSN: 0148-0227. DOI: `10.1029/2011JF002079`. (Visited on 04/04/2014).

[8] David Mohrig et al. "Interpreting avulsion process from ancient alluvial sequences: Guadalope-Matarranya system (northern Spain) and Wasatch Formation (western Colorado)". In: *Geological Society of America Bulletin* 112.12 (2000), pp. 1787–1803. ISSN: 0016-7606.

[9] Vamsi Ganti et al. "Avulsion cycles and their stratigraphic signature on an experimental backwater-controlled delta". In: *Journal of Geophysical Research: Earth Surface* 121.9 (2016), pp. 1651–1675. DOI: `https://doi.org/10.1002/2016JF003915`.

[10] Vamsi Ganti et al. “Testing morphodynamic controls on the location and frequency of river avulsions on fans versus deltas: Huanghe (Yellow River), China: Avulsion drivers on fans versus deltas”. In: *Geophysical Research Letters* 41.22 (Nov. 2014), pp. 7882–7890. ISSN: 00948276. DOI: 10.1002/2014GL061918.

[11] Liang Chen and Benjamin F. Hobbs. “Flood Control through Engineered Avulsions and Floodways in the Lower Yellow River”. In: *Journal of Water Resources Planning and Management* 146.2 (Feb. 2020). ISSN: 0733-9496, 1943-5452. DOI: 10.1061/(ASCE)WR.1943-5452.0001151. (Visited on 01/27/2020).

[12] Rudy Slingerland and Norman D. Smith. “RIVER AVULSIONS AND THEIR DEPOSITS”. In: *Annual Review of Earth and Planetary Sciences* 32.1 (May 2004), pp. 257–285. ISSN: 0084-6597, 1545-4495. DOI: 10.1146/annurev.earth.32.101802.120201. (Visited on 09/03/2014).

[13] E. A. Hajek and D. A. Edmonds. “Is river avulsion style controlled by floodplain morphodynamics?” In: *Geology* 42.3 (Mar. 2014), pp. 199–202. ISSN: 0091-7613, 1943-2682. DOI: 10.1130/G35045.1. (Visited on 04/08/2015).
